# Animal Models of Mitochondrial Diseases Associated with Nuclear Gene Mutations

**DOI:** 10.32607/actanaturae.25442

**Published:** 2023

**Authors:** O. A. Averina, S. A. Kuznetsova, O. A. Permyakov, P. V. Sergiev

**Affiliations:** Institute of Functional Genomics, Lomonosov Moscow State University, Moscow, 119991 Russian Federation; Belozersky Institute of Physico-Chemical Biology, Lomonosov Moscow State University, Moscow, 119991 Russian Federation; Department of Chemistry, Lomonosov Moscow State University, Moscow, 119991 Russian Federation

**Keywords:** mitochondrial diseases, nDNA, mutations, animal models

## Abstract

Mitochondrial diseases (MDs) associated with nuclear gene mutations are part of a large group of inherited diseases caused by the suppression of energy metabolism. These diseases are of particular interest, because nuclear genes encode not only most of the structural proteins of the oxidative phosphorylation system (OXPHOS), but also all the proteins involved in the OXPHOS protein import from the cytoplasm and their assembly in mitochondria. Defects in any of these proteins can lead to functional impairment of the respiratory chain, including dysfunction of complex I that plays a central role in cellular respiration and oxidative phosphorylation, which is the most common cause of mitopathologies. Mitochondrial diseases are characterized by an early age of onset and a progressive course and affect primarily energy-consuming tissues and organs. The treatment of MDs should be initiated as soon as possible, but the diagnosis of mitopathologies is extremely difficult because of their heterogeneity and overlapping clinical features. The molecular pathogenesis of mitochondrial diseases is investigated using animal models: i.e. animals carrying mutations causing MD symptoms in humans. The use of mutant animal models opens new opportunities in the study of genes encoding mitochondrial proteins, as well as the molecular mechanisms of mitopathology development, which is necessary for improving diagnosis and developing approaches to drug therapy. In this review, we present the most recent information on mitochondrial diseases associated with nuclear gene mutations and animal models developed to investigate them.

## INTRODUCTION

Mitochondrial diseases (MDs), which are caused by nuclear gene mutations, are a heterogeneous group of inherited diseases affecting all mitochondrial processes. Nuclear DNA (nDNA) encodes not only most of the structural proteins of the oxidative phosphorylation (OXPHOS, approximately 80 proteins) system, but also all the proteins necessary for OXPHOS protein import from the cytoplasm and their assembly in mitochondria. Defects in any of these proteins can lead to functional impairment of the respiratory chain and, therefrom, the development of MDs. Similar negative effects can also be induced by dysfunction in the proteins that control the stability and/or integrity of mitochondrial DNA (mtDNA). MDs are also caused by some disorders associated with the dysfunction of proteins from other organelles, such as WFS1, in the endoplasmic reticulum, or EIF2S3, in the cytoplasm [1, 2].

nDNA mutations associated with MDs are autosomal- dominant or recessive and can be located on the X chromosome as well. They are found in more than 300 genes, which accounts for 78.5% of the total number of genes whose mutations are associated with MDs [3, 4].

Oxidative phosphorylation provides energy to most mammalian cells and tissues. The OXPHOS system includes five multisubunit protein complexes comprising more than 80 nDNA-encoded proteins and 13 mtDNA-encoded subunits. Mutations in the individual components of OXPHOS result in a heterogeneous group of inborn errors of metabolism – primary MDs.

The most common cause of MDs is a dysfunction in complex I, the largest OXPHOS enzyme complex, which plays a central role in cellular respiration and oxidative phosphorylation [5]. Complex I deficiency symptoms most often manifest in childhood: approximately 75% of patients do not survive beyond the age of 10 years, and almost 50% of them die before the age of 2 years [6]. The first report of a mutation in an nDNA-encoded complex I subunit, causing Leigh syndrome, happened in 1998 [7]. Leigh syndrome is diagnosed in almost 80% of children with complex I deficiency, and approximately 70% of these are associated with nDNA mutations. Leigh syndrome patients experience encephalopathy, hypotonia, developmental retardation, psychomotor dysfunction, dystonia, seizures, dysphagia, respiratory dysfunction, and early mortality [8]. Complex I (NADH:ubiquinone oxidoreductase) is a 1 MDa L-shaped multiprotein complex comprising 45 different subunits arranged into six modules (N, Q, ND1, ND2, ND4, and ND5) located on hydrophobic and hydrophilic (peripheral) arms. Mutations in 39 different nuclear genes are associated with MDs caused by complex I deficiency. Most nuclear gene mutations associated with MDs are located in the NADH dehydrogenase subunit and hydrophilic arm Q-module genes. Mutations have been found both in the main catalytic subunits (NDUFV1, NDUFV2, NDUFS1, NDUFS2, NDUFS3, NDUFS7, and NDUFS8) and in the subunits responsible for complex I assembly and stability (NDUFS4, NDUFS6, NDUFA2, NDUFA12, NDUFA13, NDUFA1, NDUFA6, NDUFA8, NDUFA9, NDUFA10, NDUFA11, NDUFB3, NDUFB8, NDUFB9, NDUFB10, NDUFB11, and NDUFC2), which cumulatively are responsible for NADH:ubiquinone oxidoreductase deficiency. Destabilization of complex I also leads to several additional defects, including changes in the mitochondrial network morphology, membrane potential, and intracellular calcium homeostasis, as well as overproduction of reactive oxygen species. Data on mutations in 13 genes associated with MDs, *NDUFA5*, *NDUFAB1*, *NDUFS5*, NDUFB4,* NDUFV3*, *NDUFC1*, *NDUFB1*, *NDUFB2*, *NDUFB5*,* NDUFB6*, *NDUFAF7*, *ECSIT*, and *NDUFA3 *have not yet been obtained and/or reported. However, due to the increasing application of exome or whole-genome sequencing, new pathological mutations may be identified in the near future [3, 5].

Diagnosis of MD, which includes the assessment of serum lactate, alanine, glucose, and FGF levels, electron microscopic and histochemical analysis of mitochondrial ultrastructure, and evaluation of the enzymatic activity of OXPHOS components, is a complex task. The heterogeneous clinical manifestations of MDs complicate the achievement of a correct diagnosis and choice of an appropriate therapy. Many MD symptoms share the features of other hereditary diseases, such as diabetes mellitus, stroke, or cardiomyopathy [4]. These issues can be addressed by using animal MD models. Mutant animal models may shed light on the mechanisms that underlay MD development and the functions of the genes that encode mitochondrial proteins. Highly conserved mitochondrial proteins offer a wide array of choices of models that can be tapped to study the consequences of mutations in humans.

## ANIMAL MODELS OF MITOCHONDRIAL DISEASES

The choice of an animal MD model is always a matter of much debate. No model is inherently “good” or “bad”; the value of a particular model may only be assessed in the context of a specific project. The choice of the species, sex, and genetic properties of a model animal is based on the direction and aims of the research to achieve the most adequate transfer of the results obtained in animals to humans. It is also necessary to evaluate the possibility of using organisms from a lower step on the evolution ladder to obtain representative results without compromising their quality. The choice of a model animal should account for the factors of its availability, ease of manipulation, cost of research, ease of maintenance, and genetic and physiological homology. Genetic divergence between humans and other mammals is about 90 million years. For example, this divergence between humans and model animals such as dwarf pigs (*Porcula salvania*) and sheep (*Ovis aries*) or rabbits (*Oryctolagus cuniculus*) and rats (*Rattus norvegicus*) amounts to 94 and 87 million years, respectively [9]. For this reason, the genomes of all mammals are considered relatively similar. However, it is the laboratory mouse (*Mus musculus*) that has remained the quintessential model animal used to study human genetic MDs for many years. In general, mice and humans have almost the same set of genes. The protein-coding regions of the mouse and human genomes are approximately 85% identical. Almost every gene found in one species is identified as a closely related form in the other; in this case, some genes are 99% identical, while others are only 60% identical [10].

The first genetic studies in mice were based not on changes in the disease-modeling genotype, but on the disease-resembling phenotypes resulting from random spontaneous mutations or exposure to mutagenic factors. The advent of mouse genome editing techniques has eliminated the need to select the appropriate phenotype after random mutagenesis and enabled the generation of specific mutations in the mouse genome and the investigation of their consequences. Thus, mouse models are extremely important for elucidating the functions of genes and studying the pathological processes associated with mutations in these genes [11].

Mice with altered genomes, which model more than 50% of MDs associated with nuclear gene mutations, have been generated. However, complete inactivation of the gene under study leads to 50% embryonic lethality, although this pathological mutation can cause death at an early age [3, 4, 5, 12]. To overcome this obstacle in disease modeling, animals with heterozygous mutations in the gene of interest are examined. While some heterozygous mutants do not display a pathological phenotype, others become model animals; e.g., heterozygous *Risp*^+/P224S^ mice exhibit obviously reduced activity of mitochondrial complex III [13], and* Tfam*+/− mice are characterized by mtDNA depletion syndrome (severe decrease in mtDNA content) characteristic of people with a mutation in this gene [14]. Another approach to the investigation of vital genes is the generation of conditional tissue-specific knockouts of these genes. These knockouts have been developed for almost all lethal mutations in the nuclear genes of MDs in the tissues and organs that are most affected by each specific mutation [3, 4, 5, 12]. In the case of embryonic lethality, the ideal study object is the eggs of the *Danio rerio *fish and the *Xenopus laevis *amphibian and their fry and tadpoles, respectively [15, 16, 17]. These models have recommended themselves well in genome editing studies [18]. At early developmental stages, *D. rerio *and *X. laevis *are translucent, which enables continuous, real-time assessment and monitoring of the development of major internal organs. In this case, *D. rerio *and *X. laevis *develop independently of the parent organism and are available for direct exposure to agents at different embryogenesis stages [19]. In addition, a battery of behavioral tests has been developed to analyze neurodegenerative disorders in these animals [20, 21].

Unfortunately, there are situations when the mouse genome manipulations that should lead to MD development do not reflect the clinical picture associated with the pathological mutation in humans [22]. On the one hand, such results are associated with a higher resistance of mice to MDs [23], and on the other, some mutations in humans can manifest themselves in combination with more complex factors, such as lifestyle and concomitant diseases [24]. In these cases, a solution may be to use another animal model. For example, people carrying a mutation in the gene encoding a mitochondrial protein involved in cytochrome* c *oxidase assembly are diagnosed with Leigh syndrome, but constitutive *SURF1*–/– knockout mice do not reproduce the severity of the clinical phenotype in humans. In this case, *SURF1*–/– mutant pigs characterized by failure to thrive, muscle weakness, delayed central nervous system development in newborn piglets, and highly reduced life span become appropriate model animals [25].

It is worth noting the contribution of alternative research objects to the modeling of basic mitochondrial processes and some aspects of human pathologies:

The zebrafish *D. rerio *is considered an optimal alternative to mice. It can be used to model Leigh syndrome, in particular with liver dysfunction, and MDs involving the nervous, immune, and cardiovascular systems. Also, as mentioned above, there exist behavioral tests to assess motor and sensory response impairments typical of clinical MD manifestations [26].

The *Caenorhabditis elegans *nematode and* Drosophila melanogaster *fruit fly, despite their significant divergence from humans (686 million years [9]), have emerged as powerful genetic MD models, with great potential in early high-throughput screening for therapeutic agents [27, 28].

Microorganisms are also used to study MDs. For example, mitochondrial functions in humans and* Saccharomyces cerevisiae *(yeast) are highly similar. Yeast can be used to reproduce pathogenic mutations leading to mitochondrial dysfunction in humans. Yeast is a good alternative model for studying MDs [29] and screening therapeutic agents [30].

Therefore, the generation of reliable animal models enables us to study the functions of mitochondrial protein-encoding genes and the molecular mechanisms of MD development, which is necessary to improve diagnostics and develop approaches to drug therapy.

## ANIMAL MODELS OF HUMAN MITOCHONDRIAL DISEASES ASSOCIATED WITH NUCLEAR GENE MUTATIONS (SUMMARY TABLE)

Based on an analysis of publications devoted to various MDs [4, 5] and information gleaned from the periodically updated resources Genomics England PanelApp [3] and Online Mendelian Inheritance in Man [12], we prepared a table that summarizes the most recent data on MDs associated with nuclear

## CONCLUSION

Mitochondrial diseases are some of the most common genetic diseases. They arise at birth or develop throughout life. The genetic mutations that cause these diseases are diverse. So, researchers describing the phenotypic effect of a certain mutation have faced the problem that the mutation consequences can be mapped on a wide range of clinical manifestations. Therefore, researchers face a challenge in describing the phenotypes of mitochondrial mutations and identifying the mechanisms through which certain mutations in mitochondrial protein-coding genes manifest themselves at the level of the organism. This problem may be addressed using modeling of MDs in animals. Manipulations with the genome of model animals, in particular mice, are often able to accurately reproduce the clinical picture of human MDs, providing the opportunity to study the molecular mechanisms of pathological processes, test drugs, predict their efficacy, and select new treatment modalities.

**Table:** Animal models of human mitochondrial diseases associated with nuclear gene mutations

Gene	Clinical picture	Inheritance mode
Mitochondrial complex I deficiency		
ACAD9 acyl-CoA dehydrogenase family, member 9	• hypertrophic cardiomyopathy, • reduced exercise tolerance, • mild beta oxidation deficiency	Autosomal recessive
Acad9 knockout mice [31]: • complete Acad9 inactivation leads to embryonic lethality; • cardiac-specific Acad9 knockout is associated with the lack of expression of Acadvl (encodes long-chain acyl-CoA dehydrogenase) and Ecsit (encodes a protein involved in complex I assembly) genes in cardiac tissue; expression of Acadm, encoding medium-chain acyl-CoA dehydrogenase, is reduced; complex I dysfunction; cardiomyopathy with atrial and ventricular thickening is diagnosed by day 14 of life; • muscle tissue-specific Acad9 knockout is associated with reduced exercise tolerance and lactic acidosis.		
NDUFS1 NADH-ubiquinone oxidoreductase Fe-S protein 1	• Leigh syndrome • cavitating leukoencephalopathy	Autosomal recessive
Homozygous Ndufs1 knockout in mice is lethal [32].		
NDUFS4 NADH-ubiquinone oxidoreductase Fe-S protein 4	• combined complex I and III deficiency, • Leigh syndrome, • hypertrophic cardiomyopathy	Autosomal recessive
Complete Ndufs4 knockout mice [33, 34, 35]: • Leigh-like syndrome, • ataxia, neurological disorders, • developmental delay, • development of blindness by day 21 of life, • early mortality. Tissue-specific Ndufs4 knockout targeting neurons and glia [36]: • progressive lethal encephalopathy, • reactive glial cell phenotype, neuronal loss, ataxia; • respiratory disturbance. • Cardiac-specific Ndufs4 knockout [37, 38]: • hypertrophic cardiomyopathy. Ndufs4 point mutation [39]: • embryonic lethality of homozygous Ndufs4–/– mice, • reduced activity of complex I in heterozygous mice upon stable activity of complex II. reduced activity of complex I in heterozygous mice upon stable activity of complex II.		
NDUFS6 NADH-ubiquinone oxidoreductase Fe-S protein 6	• lactic acidosis with fatal outcome in the neonatal period, • mitochondrial encephalomyopathy, • Leigh syndrome	Autosomal recessive
Ndufs6 knockdown mice [40, 41]: • cardiomyopathy, systolic dysfunction, • renal disease associated with ultrastructural changes.		
NDUFV1 NADH-ubiquinone oxidoreductase flavo protein 1	• mitochondrial encephalomyopathy, • cerebral ataxia, • Leigh syndrome	Autosomal recessive
Transgenic Caenorhabditis elegans (soil nematode) strain with mutations in the Nuo-1 gene, the Ndufv1 homolog [42]: • lactic acidosis, • decreased NADH-dependent mitochondrial respiration, • hypersensitivity to exogenous oxidative stress.		
NUBPL nucleotide-binding protein-like protein	• leukoencephalopathy	Autosomal recessive
Homozygous Nubp1 knockout in mice is lethal [32].		
NDUFAF7 NADH dehydrogenase (ubiquinone) complex I, assembly factor 7	• pathological myopia	
Lethality of homozygous Ndufaf7 knockout mice [15]. Morpholino-mediated Ndufaf7 knockdown in D. rerio [15]: • delayed hatching time, • morphological abnormalities, • decreased complex I activity.		
NDUFS7 NADH-ubiquinone oxidoreductase Fe-S protein 7	• Leigh syndrome	Autosomal recessive
Homozygous Ndufs7 knockout in mice is lethal [32].		
NDUFA1 NADH-ubiquinone oxidoreductase subunit a1	• Leigh syndrome	X-linked recessive
Mutant mice with targeted destruction of complex I subunit mRNA, Ndufa1 [43]: • complex I deficiency, increased levels of reactive oxygen species, • optic nerve and retinal lesions.		
NDUFA13 NADH-ubiquinone oxidoreductase subunit a13 or GRIM19 gene associated with retinoid- & interferoninduced mortality 19	• encephalopathy associated with sensory deprivation, • Leigh syndrome, • thyroid cancer	Autosomal recessive
Mutant mice deficient in Grim19 [44]: • Grim19–/– embryos die by day 9.5 of embryonic development, • delayed and abnormal development of Grim19–/– blastocysts associated with abnormal mitochondrial structure and defective complex I (GRIM-19 physically resides in complex I and is responsible for its assembly), • heterozygous Grim19+/– mice lack physiological or phenotypic abnormalities.		
NDUFA8 NADH-ubiquinone oxidoreductase subunit a8	• developmental delay, • microcephaly, • epilepsy, • weight loss and failure to thrive, • speech development defects	Autosomal recessive
Homozygous Ndufa8 knockout in mice is lethal [32].		
NDUFB11 NADH-ubiquinone oxidoreductase 1 beta subcomplex, 11	• multiple congenital malformations, • microphthalmia with linear skin defects (MLS syndrome), • embryonic male lethality, • lactic acidosis, • histiocytoid cardiomyopathy, • microphthalmia, • skin defects	X-linked
Ndufb11 knockdown in Drosophila [45]: • reduced life span, • decreased metabolic rate, • complex I assembly defects, • increased lactate and pyruvate levels.		
Mitochondrial electron transport chain defects; OXPHOS disturbance		
UCP2 uncoupling protein 2	• obesity	Not found
Ucp2 knockout mice [46, 47]: • no obesity, normal response to cold or a high-fat diet, • resistant to Toxoplasma gondii infection (forms brain cysts) thanks to an increased level of reactive oxygen species in macrophages, • glucose-induced insulin secretion increases, typical of type II diabetes mellitus. Mutant mice with increased Ucp2 expression in hypocretin (orexin) neurons [48]: • these mutant mice have increased temperature of the hypothalamus, which leads to an overall decrease in body temperature by 0.3–0.5˚C, • these mutants have increased energy efficiency and a longer average life span. Ucp1 knockout mice [49]: • sensitive to cold, which indicates impaired thermoregulation, • accumulate excess fat in brown adipose tissue, but do not become fat, • Ucp1 knockout is compensated by Ucp2 that is expressed in brown epididymal fat. Ucp3 knockout mice [50]: • Ucp3 inactivation is associated with Ucp1 and Ucp2 upregulation in brown adipose tissue, • in skeletal muscles, an increased state 3/state 4 ratio due to proton leak, • in skeletal muscles, increased production of reactive oxygen species and decreased mitochondrial aconitase.		
PDHA1 pyruvate dehydrogenase, alpha-1	• impaired glycolysis–tricarboxylic acid cycle relationship, • Leigh syndrome, • deficiency of pyruvate dehydrogenase E1-alpha, • neurological dysfunction, • lactic acidosis, • growth retardation, • early mortality	X-linked dominant
Inactivation of the mouse pyruvate dehydrogenase Pdha1 gene is embryonic-lethal [51]. D. rerio mutants with visual function defects – no optokinetic response a (noa) – are deficient in dihydrolipoamide-Sacetyltransferase (Dlat), the PDH E2 subunit [16]: • a phenotype similar to pyruvate dehydrogenase complex deficiency syndrome in humans (neurological dysfunction, lactic acidosis, growth retardation, early death).		
PDSS2 renyl diphosphate synthase, subunit 2	• defect in ubiquinone (CoQ10) synthesis, • encephalomyopathy, • tubulopathy, • ataxia	Autosomal recessive
Embryonic lethality of Pdss2 knockout mice [52]. Tissue-specific Pdss2 knockout targeting glomerular podocytes [52]: • nephrotic syndrome without changes in the coenzyme Q level in kidney homogenates. Tissue-specific Pdss2 knockout targeting hepatocytes [52]: • coenzyme Q depletion in liver homogenates, • mitochondrial respiratory chain dysfunction, • disruption of basic metabolic processes. Tissue-specific Pdss2 knockout targeting the kidney (Pdss2kd/kd) [53]: • mitochondrial renal ultrastructural abnormalities, renal CoQ deficiency, decreased respiratory chain activity, increased oxidative stress, • development of nephropathy and proteinuria, up to fatal renal failure, • cerebellar abnormalities.		
COQ9 coenzyme Q9	Coenzyme Q10 (ubiquinone) deficiency	Autosomal recessive
Mice with a truncating R239X mutation in the Coq9 gene [54]: • impaired mitochondrial respiration with loss of ATP and complex I activity, • encephalomyopathy, • neuronal death, demyelination, vacuolization, spongiform degeneration, and astrogliosis, • cardiac fibrosis, • impaired locomotor activity and progressive paralysis, • early mortality.		
CYCS cytochrome C somatic isoform	• thrombocytopenia	Autosomal dominant
Cyt c deficiency in mice causes embryonic lethality and attenuates stress-induced apoptosis [55]. Mice expressing mutant cyt c (KA allele) that retains the electron transfer function, but is incapable of Apaf-1 activation [56]: • exencephaly and hydrocephalus, • cachexia, • lymphopenia.		
COQ2 coenzyme Q2, polyprenyltransferase	• encephalomyopathy, • tubulopathy, • ataxia	Autosomal recessive
	• tendency to multiple system atrophy	Autosomal recessive/dominant
Homozygous Coq2 knockout in mice is lethal [32].		
Mitochondrial complex II deficiency		
SDHD succinate dehydrogenase complex, subunit D, integral membrane protein	• paraganglioma and gastric stromal sarcoma, • pheochromocytoma	Autosomal recessive/dominant
Homozygous Sdhd knockout in mice is lethal [57, 58].		
SDHA succinate dehydrogenase complex, subunit A, flavo protein	• Leigh syndrome, • cardiomyopathy	Autosomal recessive
	• neurodegeneration and ataxia, • optic nerve atrophy, • paraganglioma	Autosomal recessive
Homozygous Sdha knockout in mice is lethal [32].		

## Abbreviations

MD: mitochondrial disease
mtDNA: mitochondrial DNA
nDNA: nuclear DNA
OXPHOS: oxidative phosphorylation
NADH: reduced form of nicotinamide adenine dinucleotide
FGF: fibroblast growth factor
